# Food Insecurity in Farta District, Northwest Ethiopia: a community based cross–sectional study

**DOI:** 10.1186/1756-0500-7-130

**Published:** 2014-03-07

**Authors:** Worku Endale, Zelalem Birhanu Mengesha, Azeb Atinafu, Akilew Awoke Adane

**Affiliations:** 1 Dera District Health Office, South Gondar Zone, Dera, Ethiopia; 2 Department of Reproductive Health, Institute of Public Health, College of Medicine and Health Sciences, University of Gondar, Gondar, Ethiopia; 3 Department of Human Nutrition, Institute of Public Health, College of Medicine and Health Sciences, University of Gondar, Gondar, Ethiopia; 4 Department of Epidemiology and Biostatistics, Institute of Public Health, College of Medicine and Health Sciences, University of Gondar, Gondar, Ethiopia

**Keywords:** Food insecurity, Prevalence, Northwest Ethiopia

## Abstract

**Background:**

Access to sufficient food is essential for household welfare as well as for accomplishing other development activities. Households with insufficient access to food often face other challenges related to food insecurity including poor health and a decline in productivity. These challenges can often create a vicious circle whereby households are unable to produce enough food even during a good crop season. Thus, this study aimed to determine the magnitude of food insecurity and its determinants in rural households of Farta District, Northwest Ethiopia.

**Methods:**

A community based cross-sectional study was conducted from September to October 2012. Household heads were recruited using a multistage random sampling technique. Data were collected by face-to-face interviews using the Household Food Insecurity Access Scale (HFIAS) tool after verbal informed consent. Data were entered to Epi info 2002 and exported to SPSS version 16 for analysis. Multiple logistic regressions were fitted and odds ratios with 95% confidence intervals were calculated to identify associated factors and control confounding effect.

**Results:**

A total of 836 households were included in this study. Nearly three quarters of the households (70.7%) had food insecurity. Households headed by females (AOR = 3.18, 95% CI:1.08, 15.21), lack of education (AOR = 2.59, 95% CI: 1.46, 4.60), family size of 4-7 (AOR = 2.39, 95% CI: 1.21,4.70), family size of >7 (AOR = 13.23,95% CI:6.18, 28.32), few or absence of livestock (AOR = 5.60, 95% CI:1.28, 24.43), absence of income from off-farm activities (AOR = 3.12, 95% CI:1.53, 6.36), lack of irrigation (AOR = 3.54, 95% CI:2.14, 5.18) and lack of perennial income (AOR = 3.15, 95% CI:1.88, 5.27) were factors associated with food insecurity.

**Conclusions:**

This study revealed that most households of the district were food insecure. Hence, the promotion of contraceptive use, off-farm employment activities and the development of small scale irrigation are important recommendations to reduce food insecurity.

## Background

Food insecurity exists when people lack access to sufficient amounts of safe and nutritious food, and therefore are not consuming enough food for an active and healthy life. This may be due to the unavailability of food, inadequate purchasing power, or inappropriate utilization at household level. About 870 million people are estimated to have been undernourished (in terms of dietary energy supply) in the period of 2010-2012. This figure represents 12.5 percent of the global population, one in eight people. The vast majority of these 852 million persons live in developing countries [1,2].

Food insecurity is a major public health problem in both developing and developed nations. However, the proportion of undernourished people remains highest in sub-Saharan Africa (239 million). Food insecurity is found to be a risk factor for poor health including chronic diseases and Human Immuno-deficiency Virus (HIV) infection [3-6].

Ethiopia, one of the most food insecure countries in Africa, has long history of famines and food shortages. More than half of the African’s food insecure population lives in Ethiopia and six other countries. The Ethiopian economy is among the most vulnerable in sub-Saharan Africa [7,8]. The number of food insecure households in Ethiopia has been increasing since the 1960s, as domestic food production has failed to meet the requirement of the country with its population growth. The annual food deficit increased from about 0.75 million tons in 1979-1980 to 1.4 million tons in 2000. It was estimated in 2004 that up to 60% of the rural and 40 percent of urban population faced the risk of food insecurity in Ethiopia [9]. In rural parts of the Ethiopia, the proportion of undernourished population in 2005 was estimated to be 45%. A total of 7.5 million chronically food insecure people received productive safety net program (PSNP) assistance in 2009 [10].

The measurement of the food insecurity level and its associated factors is an important aspect of a better targeting of high risk population groups and the establishment of reliable monitoring and evaluation systems for food security. Thus, this study aimed to determine the prevalence and associated factors of food insecurity among households of Farta District, Northwest Ethiopia.

## Methods

A community based cross-sectional survey was carried out from September to October 2012 in Farta district. Farta district is located in the Northwest part of Ethiopia and about 667 km from Addis Ababa. The area is situated at the altitude which ranges from 2000 to 2500 meters above sea level and it consists of four major agro-ecological zones: 25% low land, 45% medium highland, 24% highland and 6% gorge. The annual temperature ranges between 9 and 25 degree Celsius and the rainfall varies from around 1250 mm in the lowlands to 1500 mm in the highland areas during the rainy season. Agriculture and pastoralism (mixed) are the main economic activities in the district. According to the 2007 Ethiopian baseline census, the total population of the district was 251,321 [11].

This study included 836 households selected using a multistage random sampling technique. In the first stage, eight out of 37 rural *kebeles* (the smallest administration unit in Ethiopia) were selected by using a simple random sampling technique. Then, a total of 836 households were selected by a systematic random sampling technique. In this process, the households to be included were proportionally allocated to each selected *kebeble*. Only one individual, the identified household head, was interviewed.

Data were collected using a structured questionnaire. The questionnaire was adopted from the Household Food Insecurity Access Scale (HFIAS) measurement tool. We classified households based on the HFIAS tool into two levels of food security status i.e. food secured if the household head responded ‘no’ to all of the items and insecure if the head of the household responded to at least one ‘yes’ to items of 1-9 [12]. Data collectors were ten diploma graduate nurses and supervision were conducted by the investigators. One day training was given on interview techniques and other issues to assure quality of the data.

To quantify the livestock numbers of various species as a single figure that expresses the total amount of livestock present, the Tropical Livestock Unit (TLU) was used. The Tropical Livestock Unit is a common unit used to describe livestock numbers of various species as a single figure that expresses the total amount of livestock present–irrespective of the specific composition. A tropical livestock unit (TLU) is equivalent to 250 kilogram of live weight and refers to total livestock ownership of the household head. Each livestock of a household was changed to its equivalent TLU using conversion factors (1 cattle = 1TLU; 1 goat = 0.15 TLU; 1 horse = 1 TLU; 1 mule = 1.15 TLU; 1 donkey = 0.65 TLU; and 1 poultry = 0.005 TLU) [13].

Data were entered using Epi Info 2002 and exported to SPSS version 16 for analysis. The Hosmer-Lemeshow goodness-of-fit statistic was used to check if the necessary assumptions for multiple logistic regressions were fulfilled and the model had p-value >0.05 which suggests the model is good. Both bi-variate and multivariate logistic regressions were fitted to identify factors associated with food insecurity. All factors with a p-value <0.2 in the bi-variate logistic regression analysis were further entered into the multivariate model to control confounding effects. Both crude and adjusted odds ratios with their 95% confidence intervals (CI) were calculated and the statistical significance was accepted at the 5% level of significance (p < 0.05).

Ethical clearance was obtained from the University of Gondar, Institute of Public Health. Then, a permission letter was obtained from each *kebele* administration office. Respondents were fully informed about the purpose of the study and gave verbal consent. Households were coded by numbers rather than family names. Confidentiality of the information was assured by all the data collectors and investigators.

## Results

### Socio-demographic characteristics

A total of 836 households were included in this study. The majority (86.2%) of the household heads interviewed were men. Five hundred fifty-five (66.4%) of the respondents were in the age group of 25-50 years and the mean age was 46.45 (±12.44 SD). About 43% of households had 4-7 family members. More than one third (37.5%) of the households had access to irrigation and about one fifth (18.9%) of households had off-farm income from various other employment activities. More than two thirds (69.5%) of households possessed less than 2.5 TLU (Table 1).

**Table 1:** Socio-demographic characteristics and other assets of households in Farta district, Northwest Ethiopia, 2012

**Characteristics**		**Frequency**	**Percent**
Sex of the household head	Male	721	86.2
	Female	115	13.8
Marital status	Unmarried	8	1.0
	Married	715	85.5
	Divorced	47	5.6
	Widowed	54	6.5
	Separated	12	1.4
Religion	Orthodox	834	99.8
	Muslim	2	0.2
Family size	1-3	163	19.5
	4-7	359	42.9
	>7	314	37.6
Education level	Can’t read and write	654	78.2
	Can read and write	182	21.8
Occupation	Farmer	796	95.2
	Merchant	27	3.2
	Daily laborer	13	1.6
Land ownership	No land	20	2.4
	<0.5 hectare	420	50.2
	0.5-1 hectare	329	39.3
	>1 hectare	67	8.1
Access to irrigation	Yes	311	37.2
	No	525	62.8
Perennial plants	Yes	338	40.4
	No	498	59.6
TLU	<2.5	581	69.5
	≥2.5	255	30.5
Off-farm activities	Yes	158	18.9
	No	678	81.1
Crop production	<8.85 quintal	472	56.6
	>8.85 quintal	362	43.4

TLU-Total Livestock Unit.

### Prevalence of food insecurity

A high proportion (67.6%) of the heads of the households had worries about the availability of enough food for their family. Similar proportions of the household heads (68.3%) reported the absence of the preferred food to eat and 66.7% of respondents reported that they consumed a limited variety of food. The overall prevalence of food insecurity was 70.7% (Table 2).

**Table 2:** The household food insecurity status in Farta district, Northwest Ethiopia, 2012

**Character**		**Frequency**	**Percentage**
Worry about not having enough food	Yes	565	67.6
	No	271	32.4
Unable to eat preferred food	Yes	571	68.3
	No	265	31.7
Eat just a few kinds of food	Yes	558	66.7
	No	278	33.3
Eat food really do not want	Yes	210	25.1
	No	626	74.9
Eat smaller amounts in meal	Yes	462	55.3
	No	374	44.7
Eat fewer meals in a day	Yes	442	52.9
	No	394	47.1
No food of any kind in household	Yes	36	4.3
	No	800	95.7
Go to sleep hungry	Yes	387	46.3
	No	449	53.7
Go a whole day & night without food	Yes	17	2.0
	No	819	98.0

### Factors associated with food insecurity

In multivariate logistic regression analysis, almost all variables like being a female household head, having a large family size, lack of education of the household head, a lack of access to irrigation, lack of income from perennial and off-farm activities and having few or no livestock were significantly and independently associated with food insecurity.

In this study, households headed by females were about 3 times (AOR = 3.18, 95% CI: 1.08, 15.21) more likely to be food insecure. Households with family sizes of 4-7 were 2 (AOR = 2.39, 95% CI: 1.21, 4.70) times more likely to be food insecure when compared to families with smaller (1-3) family sizes. Similarly, households with more than 7 family members were about 13 times (AOR = 13.23, 95% CI: 6.18, 28.32) more likely to be food insecure. On the other hand, household heads who were unable to read and write were more than 2.5 times more likely to be food insecure when compared with household heads who could read and write (AOR = 2.59, 95%CI:1.46, 4.60). Access to irrigation was also the other factor significantly associated with food insecurity. Households that had no access to irrigation were about 3.5 times more likely to be food insecure than households that had access to irrigation (AOR = 3.54, 95% CI: 2.14, 5.18). Similarly, households that did not practice off-farm activities were about 3 (AOR = 3.12, 95% CI: 1.53, 6.36) times more likely to be food insecure (Table 3).

**Table 3:** Logistic regression analysis of factors associated with food insecurity in households of Farta district, Northwest Ethiopia, 2012

**Characteristics**		**Food security status**		**Crude OR**	**Adjusted OR**
		**Unsecured**	**Secured**	**(95% C.I)**	**(95% C.I)**
Sex of household heads	Male	485	236	1	1
	Female	106	9	5.73(2.85,11.52)	3.18(1.08,15.21)
Family size	1-3	90	73	1	1
	4-7	235	124	1.54(1.05,2.24)	2.39(1.21,4.70)
	>7	266	48	4.49(2.91,6.95)	13.23(6.18,28.32)
Education	Illiterate	503	151	3.56(2.52,5.01)	2.59(1.46,4.60)
	Literate	88	94	1	1
Livestock possession	1-5	426	110	19.36(6.48,57.6)	5.60(1.28,24.43)
	6-10	117	114	5.13(1.70,15.48)	2.48(0.56,10.47)
	>10	4	20	1	1
Off-farm activities	Yes	52	106	1	1
	No	539	139	7.90(5.40,11.57)	3.12(1.53,6.36)
Irrigation	Yes	142	169	1	1
	No	449	76	7.03(5.05,9.78)	3.54(2.14,5.18)
Perennial plants	Yes	172	192	1	1
	No	398	53	8.82(6.21,12.55)	3.15(1.88,5.27)

## Discussion

Recently, food insecurity has received increased attention everywhere because of the worsening global economic conditions. In addition to affecting dietary intake, food insecurity and hunger ultimately impact nutritional status, and physical and mental well-beings of people [3,14]. Food insecurity is also a potential risk factor associated with chronic diseases [5].

Food insecurity is one of the most crucial problem threatening millions of people in Ethiopia. The HFIAS scale measurement revealed that 70.7% of the households faced food insecurity which was higher than findings reported from Sidama, Southern Ethiopia (54.1%) and Addis Ababa (58%) [15,16]. The possible justifications might be seasonality, geographical and rainfall variations. Since these data were collected in September, seasonality and food insecurity might be linked. In most parts of Ethiopia, September is a transition from the rainy to the harvest season and deterioration of food storage is common in this month. Similarly, this difference might be attributable to geographical and rainfall variations. In northern Ethiopia, where this study was conducted, the rainy season is only from June to August unlike Southern Ethiopia.

This study also elicited responses showing the extent to which food was a serious concern in the households. For instance, the responses to the simple emotional insecurity question (i.e. worrying about food) showed that 67.6% reported that worrying about the sources and amount of food their family members would have in the days to come and 68.3% of the respondents were unable to eat the culturally accepted ‘standard’ amount and quality of food due to the decline in food access.

In Ethiopia, most resources including land are owned by males, and women have less access to education and other assets. This study showed that households headed by females were about three times more likely to be food insecure than households headed by males. This finding is supported by a study done in Nigeria [17]. Even when they had an adequate number of oxen and farm land, female headed households were still more likely to be food insecure as women could not plow their land as men could do in a timely manner. Plowing land is also traditionally given only to males. As a result, women need men’s labor to plow their land in exchange for other expenses such as cash and crops. This consequently reduced their income and compromised their status of food security. A population-based study among adolescents living in South-western Ethiopia using experienced-based scales also indicated that girls were more likely than boys to report being food insecure themselves [18].

Large family size tends to exert more pressure on food consumption than the labor it contributes to production. From this study, we observed that the larger the family size, the more likely the family was food insecure. This can be explained by the fact that the majority of farmers residing in Ethiopia are small scale semi-subsistence producers with limited land size and limited non-agricultural activities. A study conducted in Nigeria supports this finding [19].

Educational attainment by the household heads could lead to awareness of possible advantages of modernized agriculture by means of technological inputs; enable them to read instructions on fertilizer packs and diversification of household incomes which, in turn, would enhance the household’s food supply. In this study, it was found that household head’s educational status was shown to have a statistically significant association with food insecurity. Households whose heads could not read and write were more likely to be food insecure when compared with households whose heads were able to read and write. Studies done in Sidama district, Southern Ethiopia and Bangladesh [20,21] also supported the above conclusion.

Perennial resources are important variables to reduce food insecurity of households by different mechanisms: fruit and vegetable are important components of a healthy diet and, if consumed daily in sufficient amounts, can help to prevent major diseases and to improve health and may play an important role in poverty reduction. They can also provide employment opportunities and sources of income. Households that had no perennial resource income were more likely to be food insecure than households that had income from these resources.

The other important determinants of household food insecurity in the study area were the number of livestock owned and off-farm activities. Livestock possession enables the households to be food secure either through the income earned or by direct consumption. Households with no or few numbers of livestock were more likely to be food insecure. Off-farm activities also enhance the household economy and food security by giving additional income and decreases food deficits when agricultural production falls short and it also avoids the sale of their grain. Households that had no off-farm activities were more likely to be food insecure than households that had.

This study focused on determining the magnitude of food insecurity and identifying factors that influence household food insecurity in the rural areas of Farta district. However, some of the variables believed to affect food insecurity are not addressed by this research. These variables include, for example, climatic and weather conditions, rainfall and temperature, topography, natural disasters and ecological conditions that require further research.

## Conclusion

A high proportion of households were food insecure. Besides this, households headed by females, a lack of education, family sizes of 4-7 and above 7, few or the absence of livestock, lack of off-farm activities, lack of irrigation, and lack of perennial income were associated factors with food insecurity. Hence, promotion of education, contraceptive use, off-farm activities and the development of small scale irrigation project are important recommendations to reduce food insecurity.

## Competing interests

The authors declare that they have no conflict of interests.

## Authors’ contributions

All authors designed the study, performed the statistical analysis, and drafted the paper. All authors read and approved the manuscript.
